# 29-Color Flow Cytometry: Unraveling Human Liver NK Cell Repertoire Diversity

**DOI:** 10.3389/fimmu.2019.02692

**Published:** 2019-11-19

**Authors:** Iva Filipovic, Isabella Sönnerborg, Benedikt Strunz, Danielle Friberg, Martin Cornillet, Laura Hertwig, Martin A. Ivarsson, Niklas K. Björkström

**Affiliations:** ^1^Department of Medicine Huddinge, Center for Infectious Medicine, Karolinska Institute, Karolinska University Hospital, Stockholm, Sweden; ^2^Division of Transplantation Surgery, Department of Clinical Science, Intervention and Technology, Karolinska Institute, Stockholm, Sweden; ^3^Department of Surgical Sciences, Uppsala University, Uppsala, Sweden

**Keywords:** natural killer cells, liver immunology, tissue-resident cells, high-dimensional, flow cytometry

## Abstract

Recent studies have demonstrated extraordinary diversity in peripheral blood human natural killer (NK) cells and have suggested environmental control of receptor expression patterns on distinct subsets of NK cells. However, tissue localization may influence NK cell differentiation to an even higher extent and less is known about the receptor repertoire of human tissue-resident NK cells. Advances in single-cell technologies have allowed higher resolution studies of these cells. Here, the power of high-dimensional flow cytometry was harnessed to unravel the complexity of NK cell repertoire diversity in liver since recent studies had indicated high heterogeneity within liver NK cells. A 29-color flow cytometry panel allowing simultaneous measurement of surface tissue-residency markers, activating and inhibitory receptors, differentiation markers, chemokine receptors, and transcription factors was established. This panel was applied to lymphocytes across three tissues (liver, peripheral blood, and tonsil) with different distribution of distinct NK cell subsets. Dimensionality reduction of this data ordered events according to their lineage, rather than tissue of origin. Notably, narrowing the scope of the analysis to the NK cell lineage in liver and peripheral blood separated subsets according to tissue, enabling phenotypic characterization of NK cell subpopulations in individual tissues. Such dimensionality reduction, coupled with a clustering algorithm, identified CD49e as the preferred marker for future studies of liver-resident NK cell subsets. We present a robust approach for diversity profiling of tissue-resident NK cells that can be applied in various homeostatic and pathological conditions such as reproduction, infection, and cancer.

## Introduction

The last five decades have seen extraordinary developments in the understanding of natural killer (NK) cell biology. NK cells are innate lymphocytes originally discovered as cells capable of killing tumor cells and later virally-infected cells (1, 2). One of the major pathways of cell death mediated by NK cells involves secretion of cytolytic molecules like perforin and granzymes, which makes NK cells functionally related to their adaptive counterpart, cytotoxic T lymphocytes (CTLs) (3, 4). However, the mechanisms which trigger the killing of the target cells are fundamentally different between these two lineages. NK cells use an array of germline-encoded receptors to carry out their main tasks associated with the recognition of non-self: tumor surveillance and clearance of viral infections (5, 6). Engagement of distinct activating and inhibitory receptors expressed on the surface of NK cells by their respective ligands determines the functional response. Importantly, genetic and environmental determinants shape the overall diversity of these receptors (7).

Since their discovery, it has become clear that NK cells are found not only in circulation, but also in lymphoid organs as well as non-lymphoid organs like uterus and liver (8). The liver is instrumental in regulating systemic homeostasis, and represents an organ with a dynamically changing microenvironment (9). Notably, it is also highly enriched in immune cells and has a distinct immune composition: NK cells are among the most abundant, representing 30–40% of human intrahepatic lymphocytes compared to the 10–15% typically observed in peripheral blood (10). The microenvironment of the liver has a complex anatomical organization (11) and is essential in maintaining tolerance toward antigens derived from the gut, including the diverse gut microbiome, via the gut-liver axis (12). Unsurprisingly, a subset of liver NK cells with antigen-specific memory was described in the mouse (13). These cells express CXCR6 which, although not required for antigen recognition, reliably labels this subset of liver NK cells in mouse, but also a subset residing in human liver (14). Similarly, mouse parabiosis studies demonstrating existence of liver-resident CD49a^+^ NK cells (15) led to the first characterization of a human counterpart (16). Other studies have shown that liver Eomes^hi^T-bet^lo^ NK cells are absent from blood but also that they do not overlap entirely with previously identified CD49a^+^ subset (14, 16–18). Yet another report, using cytometry by time-of-flight (CyTOF) followed by flow cytometry validation, identified for the first time CD49e^−^ NK cells as the human liver-resident NK cell population (19). Altogether, this suggests an underlying heterogeneity within liver NK cell subsets.

Given the limited extent to which tissue residency in human liver samples can be investigated compared to mouse models, and given the clinical implications for immune responses such as tolerance and disease, detailed phenotypic characterization of human liver NK cells is essential. One of the main challenges in reaching a consensus when comparing literature on liver NK cells comes from a limited number of markers one could analyze by conventional flow cytometry. To overcome this, we here designed a 29-color tissue NK cell-focused panel, demonstrated its potential on liver, peripheral blood and secondary lymphoid tissue, and performed deep profiling of liver NK cell diversity in comparison to peripheral blood NK cells.

## Materials and Methods

### Human Samples

Blood samples used in this study were peripheral blood mononuclear cells (PBMCs) derived from buffy coats from blood donations of healthy human volunteers from the local hospital blood bank. Liver samples were obtained from human adult liver tissue during resection surgery for primary or secondary liver malignancies. Human pediatric and adult uninfected tonsils were obtained from patients undergoing tonsillectomy due to sleep-disordered breathing or obstructive sleep apnea syndrome. All samples were from Karolinska University Hospital, Huddinge, Sweden. None of the samples were matched. All blood and tissue donors gave oral and written informed consent conforming to the provisions of the Declaration of Helsinki. The regional Ethics Committee in Stockholm, Sweden, approved all the protocols involving collection of blood, liver, and tonsil samples.

### Isolation of Peripheral Blood Mononuclear Cells

Peripheral blood mononuclear cells (PBMCs) were isolated from buffy coats using density gradient centrifugation. The blood was diluted with Phosphate Buffered Saline (PBS, Sigma) and layered onto the Ficoll-Hypaque media solution (GE Healthcare). Samples were centrifuged at room temperature, with brakes turned off, for 20 min at 2,000 revolutions per minute (rpm). The mononuclear cell layer was carefully removed from the interface and washed twice with PBS. Cells were frozen in CoolCell containers (Corning) in heat-inactivated Fetal Bovine Serum (FBS; Sigma) supplemented with 10% dimethyl sulfoxide (DMSO; Sigma) and stored in liquid nitrogen until use.

### Tissue Dissociation and Cell Isolation

Mononuclear liver cells were isolated from the tumor non-affected area of the liver tissue as previously described (20). In brief, the tissue underwent a series of flushing steps to remove excess sinusoidal blood, followed by a three-step perfusion protocol in which the final step involved enzymatic processing (with collagenase XI, Sigma). Supernatant obtained through these steps was washed and layered onto the Ficoll-Hypaque media solution for the density gradient centrifugation to isolate leukocytes in the same way as PBMCs. Whole tonsils were mechanically processed by cutting and passing through a 100 μm strainer, followed by a 40 μm straining step, and finally a density gradient centrifugation in the same way as liver and blood samples. Post-isolation, cells from liver and tonsil were frozen in FBS supplemented with 10% DMSO and stored in liquid nitrogen, similar to PBMC.

### Flow Cytometry

Vials with cryopreserved mononuclear cell suspensions isolated from peripheral blood, liver, and tonsil were thawed rapidly in a water bath at 37°C, and transferred carefully to complete cell medium (RPMI with 10% FBS, L-glutamine, Penicillin/Streptomycin). After two washes, cells were resuspended in FACS buffer (PBS with 2 mM EDTA and 2% FBS), filtered through a 40 μm strainer (BD Falcon), counted and stained immediately in 96-well V-bottom plates. All staining steps were performed at room temperature in the dark and all washing steps were performed by centrifuging plates for 2 min at 1,800 rpm at room temperature, unless otherwise stated. Cells were incubated with antibodies against surface antigens diluted accordingly in 50 μl FACS buffer for 20 min (see Table 1 for dilution details) followed by two washes with 150–200 μl FACS buffer. In the second staining step cells were stained with the LIVE/DEAD Fixable Aqua Dead Cell Stain (Thermo Fisher) and fluorescently conjugated streptavidin for 20 min. This was again followed by two washes. Next, samples were fixed for 45 min in freshly prepared fixation/permeabilization working solution from eBioscience Foxp3/Transcription Factor Staining Buffer set (Thermo Fisher). Fixing solution was removed by centrifugation and washing once in 1× permeabilization buffer from the same fix/perm kit. Finally, cells were stained with antibodies against intracellular antigens diluted in 1× permeabilization buffer from the same set for 30 min. Samples were then washed twice in 1× permeabilization buffer and resuspended in 200 μl FACS buffer. To remove potential clumps in the cell suspension, the cells were transferred into 5 ml polystyrene round-bottom tubes (BD Falcon) through the 35 μm strainer cap. The cells were acquired on a FACSymphony A5 instrument (BD Biosciences). Importantly, in all three steps where fluorescently conjugated antibodies were added, BD Horizon Brilliant Stain Buffer Plus (BD Biosciences) was supplemented at 1:5 to minimize staining artifacts commonly observed when several BD Horizon Brilliant dyes are used. Single-stained UltraComp eBeads Compensation Beads (Thermo Fisher) were used according to manufacturer's instructions to prepare compensation controls by incubating with fluorescently conjugated antibodies used in experiments. The FACSymphony A5 flow cytometer used in this study was equipped with the following lasers: UV (355 nm), violet (405 nm), blue (488 nm), yellow/green (561 nm), and red laser (637 nm). The yellow/green, blue, and violet lasers were tuned at 200 mW, the red laser was tuned at 140 mW, and the UV laser was tuned at 60 mW. An instrument cleaning program and FACSDiva Cytometer Setup and Tracking (CST) software were run daily with the CST beads, to ensure optimal cytometer performance. PMT voltages were automatically updated by applying previously created “application setting” for this study. This allowed for a rigorous and reproducible approach to panel optimization. Further information on individual filters and cytometer configuration, can be found in Table 1, in addition to details of antibodies used in this study.

**Table 1:** Antibodies used in this study.

**Antigen**	**Clone**	**Fluorophore**	**Laser line**	**BD FACSymphony filter**	**Dilution used**	**Custom conjugate**	**Company**	**Function**
CCR5	2D7/CCR5	BUV395		379/28	25	No	BD biosciences	Cell trafficking
CD16	3G8	BUV496		515/30	200	No	BD biosciences	NK cell subsets
CD56	NCAM16.2	BUV563	UV (355 nm)	580/20	200	No	BD biosciences	NK cell subsets
CD49a	SR84	BUV615		605/20	25	Yes	BD biosciences	Tissue residency/cell retention
CD38	HIT2	BUV661		670/25	25	No	BD biosciences	Maturation/activation
CD69	FN50	BUV737		735/30	50	No	BD biosciences	Tissue residency/cell retention/activation
CD45	HI30	BUV805		810/40	100	No	BD biosciences	Common lymphoid identity
CD49e	REA686	VioBright FITC		530/30	100	No	Miltenyi biotec	Tissue residency/cell retention
NKG2C	REA205	Biotin		N/A	100	No	Miltenyi biotec	Activating receptor
Streptavidin	N/A	BB630		610/20	400	Yes	BD biosciences	N/A
CD103	Ber-Act8	BB660	Blue (488 nm)	670/30	50	Yes	BD biosciences	Tissue residency/cell retention
NKG2A	131411	BB700		710/50	25	No	BD biosciences	Inhibitory receptor
Perforin	δG9	BB755		750/30	200	Yes	BD biosciences	Effector function/cytotoxicity potential
Granzyme B	GB11	BB790		810/40	100	Yes	BD biosciences	Effector function/cytotoxicity potential
Eomes	WD1928	eFluor 660		670/30	25	No	Thermo Fisher	Transcription factor
Ki-67	B56	AF700	Red (637 nm)	730/45	100	No	BD biosciences	Proliferation marker
CD57	TB03	APC-Vio770		780/60	50	No	Miltenyi Biotec	Maturation
Tim-3	7D3	BV421		450/50	50	No	BD biosciences	Co-inhibitory receptor/immune checkpoint
CD14	M5E2	V500		525/50	100	No	BD biosciences	Non-NK cell lineage exclusion
CD19	SJ25C1	BV510		525/50	100	No	BD biosciences	Non-NK cell lineage exclusion
CD123	6H6	BV510		525/50	50	No	Biolegend	Non-NK cell lineage exclusion
LIVE/DEAD Dead Cell Stain	N/A	Fixable Aqua		525/50	100	No	Thermo fisher	Exclusion of dead cells
CD8	RPA-T8	BV570	Violet (405 nm)	586/15	50	No	Biolegend	T cell subsets
CD161	DX12	BV605		605/40	25	No	BD biosciences	Maturation/NK cell subsets
CX3CR1	2A9-1	BV650		677/20	50	No	Biolegend	Cell trafficking
CXCR6	13B 1E5	BV711		710/50	50	No	BD biosciences	Cell trafficking
CD3	SK7	BV750		750/30	100	No	Biolegend	Non-NK cell lineage exclusion/T cell subsets
NKp46	9E2/NKp46	BV786		810/40	25	No	BD biosciences	Activating receptor
PLZF	R17-809	PE		586/15	50	No	BD biosciences	Transcription factor
T-bet	4B10	PE-Dazzle 594		610/20	25	No	Biolegend	Transcription factor
CD4	OKT4	PE-Cy5		670/30	200	No	Biolegend	T cell subsets
KIR2DL2, KIR2DL3, KIR2DS2	GL183	PE-Cy5.5	Yellow-green (561 nm)	710/50	50	No	Beckman coulter	Activating and inhibitory receptors
KIR2DL1, KIR2DS1	EB6	PE-Cy5.5		710/50	50	No	Beckman coulter	Activating and inhibitory receptors
CD127	A019D5	PE-Cy7		780/60	50	No	Biolegend	T cell subsets/innate lymphoid cells
Brilliant Stain Buffer Plus	N/A	N/A	N/A	N/A	5	N/A	BD biosciences	N/A

*Color-coding indicates different lasers and their colors*.

### Flow Cytometry Analysis

**Figure 1:**
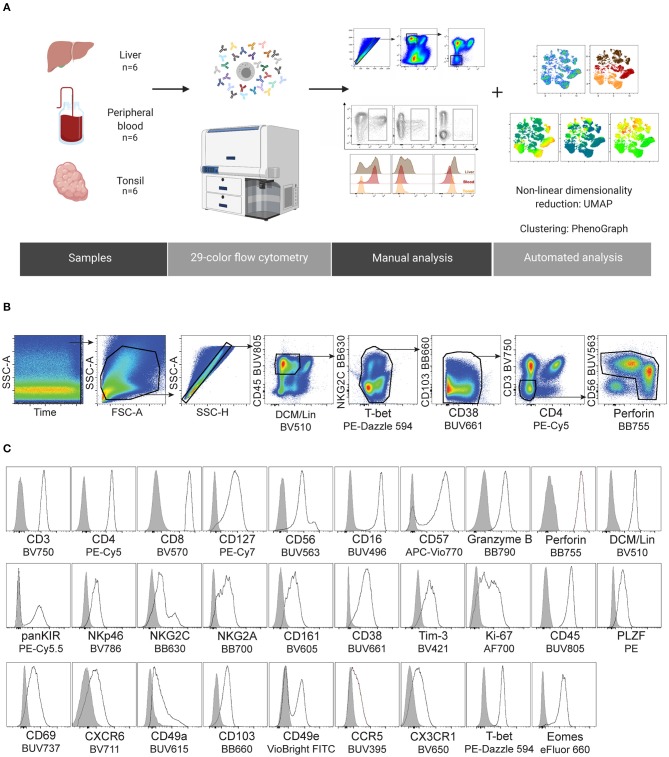
Design of a 29-color human NK cell-focused flow cytometry panel. **(A)** Summary of the experimental workflow. **(B)** Gating strategy used for identification of NK cells and downstream analysis. Two clean-up steps were performed (NKG2C BB630 vs. T-bet PE-Dazzle 594 and CD103 BB660 vs. CD38 BUV661) to remove fluorochrome aggregates. **(C)** Representative histograms for the indicated proteins (black line), including an internal negative control for each (gray shaded histogram). DCM, Dead Cell Marker; Lineage (Lin), CD14/CD19/CD123.

After acquisition on FACSymphony A5 flow cytometer, FCS3.0 files were exported from the BD FACSDiva software and imported into FlowJo v.10.6.0 (BD Biosciences). Automated compensation was calculated by FACSDiva software using single-stained compensation beads. This 29-color compensation matrix was analyzed in detail in FlowJo through investigating N-by-N view feature as well as the pairwise expression of all proteins stained for in this study. Fluorescence minus one (FMO) experiments were run prior to this study, which also aided the optimization of the compensation matrix. Based on this, the compensation matrix was adjusted where necessary due to over- or under-compensation by the automated algorithm. After the compensation matrix was adjusted, samples were concatenated and analyzed using FlowJo plugins (https://flowjo.com/exchange/#/), namely: Downsample (v.3.0.0), UMAP (v2.2), and PhenoGraph (v.0.2.1). UMAP was run using the default settings (Euclidean distance function, nearest neighbors: 15 and minimum distance: 0.5). PhenoGraph was run using the default number of nearest neighbors (*K* = 30). Parameters for running UMAP and PhenoGraph were selected depending on the experimental question and are specified in the accompanying text and figure legends. Graphs were made in Prism 8, v8.2.0 (GraphPad Software Inc.). Figure 1A was prepared in BioRender and all figures were put together in Illustrator CC 2019 (Adobe).

## Results

### Design of a 29-Color Human NK Cell-Focused Flow Cytometry Panel

NK cells in all tissues are classified as CD56^high^CD16^−^ and CD56^low^CD16^+^ NK cells, commonly referred to as CD56^bright^ and CD56^dim^ NK cells, respectively (8). These subsets of NK cells are identified both in circulation and in the liver but in different frequencies within total NK cells. Peripheral blood is rich in the CD56^dim^ population and there is generally a lower percentage of circulating CD56^bright^ NK cells. Contrasting this the liver is rich in the CD56^bright^ NK cell subset, similarly to other non-lymphoid (e.g., uterus) and secondary lymphoid organs (e.g., tonsils). When found outside of circulation, the CD56^bright^CD16^−^ NK cell population is typically considered to be the tissue-resident population (8). Yet, with respect to human liver, and as alluded to in the introduction, the tissue-resident NK cell population within this organ has been defined in multiple distinct ways suggesting a high degree of heterogeneity among these cells. This was a strong rationale for the current study, where we aimed to compare the identification of liver NK cells from different published reports.

We harnessed the power of technical advances within high-end flow cytometry and designed a comprehensive 29-color NK cell-focused flow cytometry panel to compare the diversity of tissue-resident and circulating NK cells. As a starting point, this was applied to NK cells from three tissue types to demonstrate its potential: liver, peripheral blood, and tonsil. Details of the antibodies used in panel design can be found in Table 1. We carefully considered all aspects of panel design when selecting fluorochromes for distinct antibodies (21). These considerations included: (1) titration of every antibody used in the panel, (2) application of appropriate fluorescence minus one (FMO) and isotype controls to aid in detecting fluorochrome aggregates and setting accurate positive gates, (3) alignment of the fluorochrome brightness with the antigen expression density within a cell, and (4) avoiding, when possible, high spectral overlap between fluorochromes on co-expressed markers. In total, we used 32 antibodies, in addition to the dead cell marker (DCM), to detect 29 fluorescent parameters. The focus of the panel were surface and intracellular proteins associated with tissue residency as well as those describing the functional potential of an NK cell (activating and inhibitory receptors, effector proteins, activation and differentiation markers, chemotaxis, and proliferation). The panel was designed to exclude main myeloid lineages and B cells (Lin channel: DCM, CD14, CD19, CD123) from future analysis. Since tissue residence is not only a property of NK cells and resident T cells display similar phenotypes (22), we assigned separate fluorophores to main T cell subsets to allow for relevant comparisons. Cryopreserved cells from non-matched liver, peripheral blood, and tonsil donors were stained with this 29-color panel and acquired flow cytometry data were then processed and analyzed (Figure 1A). After optimizing compensation (see section Materials and Methods), two “clean-up gates” were included in the gating strategy to remove super-fluorescent fluorochrome aggregates (Figure 1B). It is important to note that the addition of a specific buffer drastically decreased the amount of these aggregates (see section Materials and Methods). Moreover, their presence was sample-dependent and likely due to differences in quality when samples were isolated and frozen. Finally, we observed that populations with low protein expression levels for a particular antigen could be successfully distinguished from negative populations, which validated the usefulness and efficacy of our panel (Figure 1C).

### Distinct T Cell, ILC, and NK Cell Clusters Are Robustly Separated by Non-linear Dimensionality Reduction

**Figure 2:**
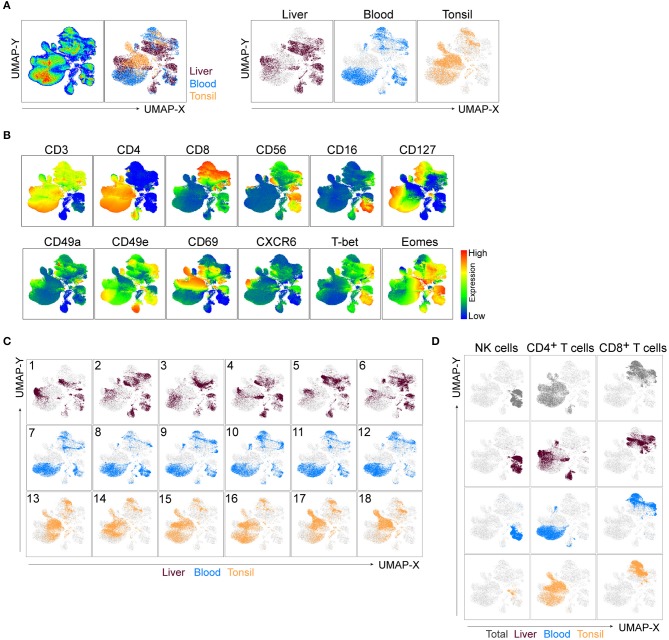
UMAP robustly embeds CD45^+^ cells from distinct tissues and identifies main clusters of lymphocytes. **(A)** UMAP plot of CD45^+^ cells. Live, Lin^−^CD45^+^ cells from liver, blood, and tonsil tissue were gated (Figure 1B), down-sampled to 25,000 cells per sample which were barcoded and concatenated. Eighteen samples were included in the analysis (six per source material). CD45 and DCM/Lin were excluded from the list of UMAP running parameters. The resulting UMAP projection is colored according to the tissue of origin (combined in left panel or individually in right panel). **(B)** UMAP plots showing expression intensities of lineage markers (top row), as well as some of the markers distinguishing liver-enriched NK cells from peripheral blood population (bottom row). See Supplementary Figure 1A for compiled plots of all other parameters. **(C)** UMAP embeddings from **(A)**, colored by the tissue of origin and displayed for each individual donor, labeled 1-18. **(D)** Events in the UMAP embeddings were overlaid with manually gated NK cells, CD4^+^, and CD8^+^ T cells and displayed for all tissues in the concatenated file (top row), or for each tissue separately (following three rows). Representative gates can be found in Supplementary Figure 1B.

To capture the non-linear structure of our single cell data, we performed dimensionality reduction using a FlowJo implementation of the recently developed uniform manifold approximation and projection (UMAP) algorithm (23). UMAP was performed on live CD45^+^ cells (gated as single, live, Lin^−^CD45^+^ cells, Figure 1B). CD45^+^ cells from individual samples were down-sampled to 25,000 events per sample, individual samples were electronically barcoded, and finally concatenated for downstream analyses. A total of 18 samples were included in the analysis, six for each source material. UMAP was run using all compensated parameters except the previously gated CD45 and DCM/Lin. Several clusters were identified in the resulting UMAP maps. These were pulled together predominantly according to the defining lineage markers rather than the tissue of origin (Figures 2A,B). All clusters contained populations found in liver, peripheral blood, and tonsil or the combination of the two, apart from one cluster which appeared to be liver-specific (Figures 2A,B). To determine what defined these clusters, we analyzed the expression of lineage markers displayed on the UMAP coordinates. There were two clearly separated clusters of CD3^+^ cells, one uniformly co-expressing CD4, and the other one with more variable levels of CD8. The IL-7 receptor (CD127) was variably expressed in both of these two clusters. It was also highly expressed in a small CD3^−^ cluster close to T cells (Figure 2B, top row), suggesting that these were innate lymphoid cells (ILCs). Furthermore, the cluster located in close proximity to the ILCs was characterized by a high expression of CD56 and absence of CD3. Within this cluster, a sub-cluster was CD16^high^, indicating that it may contain CD56^dim^ (and possibly CD56^bright^CD16^+^) NK cells. We color-mapped UMAP plots by the remaining (NK-focused) parameters in our panel, which validated our notion that the CD3^−^CD56^+^ UMAP cluster contained NK cells (Figure 2B, bottom row and Supplementary Figure 1A). The above-mentioned liver-specific cluster localized within the CD3^−^CD56^+^ UMAP cluster and was shown to contain cells expressing high levels of CD49a, CD69, CXCR6, and Eomes compared to other CD56-positive cells, as well as low expression of T-bet and CD49e (Figure 2B, bottom row).

Through manual gating analysis during panel optimization for this study, we observed a degree of donor-to-donor variability, particularly in the expression of tissue residency markers (data not shown). We tested the overall robustness of UMAP, as well as how successful it was in detecting such variability through three different approaches. Firstly, we deconvoluted individual donor samples in the concatenated file and displayed them on the UMAP embeddings of CD45^+^ cells (Figure 2C). Liver samples 3 and 5, for example, had sections of the liver-specific cluster missing, while the other CD56^+^CD16^+^CD3^−^ UMAP cluster demonstrated an even higher level of variability between non-matched blood and liver donors (Figure 2C). Secondly, we performed manual flow cytometry gating for NK cells, CD4^+^ and CD8^+^ T cells in the concatenated file in all tissues as well as in each individual tissue and overlaid cells from this analysis on the UMAP map (Figure 2D, Supplementary Figure 1B). Manually gated subsets shared the UMAP coordinates with automatically detected clusters across all tissues. Thirdly, given the focus on NK cells in our panel, we ran a UMAP analysis similar to the one in Figure 2A excluding CD56 as a clustering parameter. Reassuringly, the combined expression of all other parameters from the panel was specific enough to identify T cells and NK cells, resulting in nearly identical clustering (Supplementary Figure 1C).

### PhenoGraph Distinguishes Populations of Tissue-Enriched Lymphocytes and Their Diversity Across Individuals

**Figure 3:**
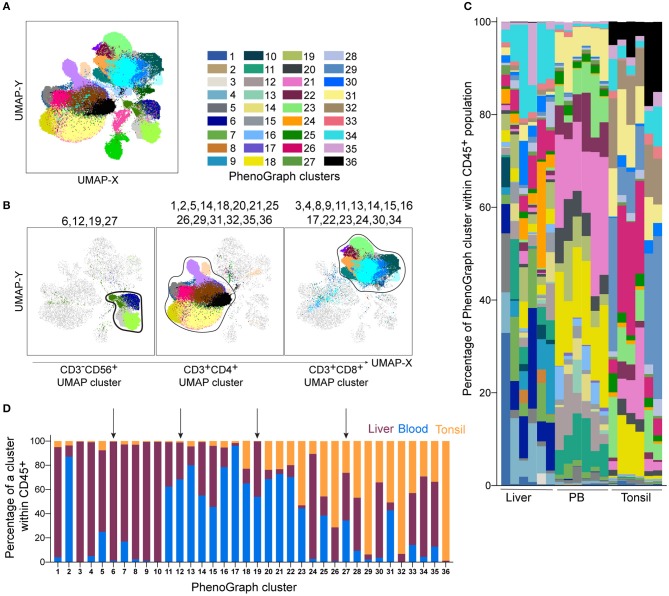
PhenoGraph analysis of the Lin^−^CD45^+^ population identifies tissue-enriched clusters and demonstrates their diversity across individuals. PhenoGraph clustering was performed on Lin^−^CD45^+^ barcoded and concatenated cells from all samples, CD45 and DCM/Lin were excluded from the list of running parameters. **(A)** Plot of all 36 identified PhenoGraph clusters overlaid on the UMAP projection. **(B)** Selected PhenoGraph clusters displayed over UMAP embeddings. Previously identified main lineage UMAP clusters are indicated by black lines (CD3^−^CD56^+^ cluster, CD3^+^CD4^+^ and CD3^+^CD8^+^ cluster). **(C)** Stacked bars showing relative abundance of every PhenoGraph cluster within CD45^+^ cells in each liver, peripheral blood (PB), and tonsil sample. Color coding same as in **(A)**. **(D)** Relative abundance of liver, blood, and tonsil CD45^+^ cells within each detected PhenoGraph cluster. Arrows indicate PhenoGraph clusters within the CD3^−^CD56^+^ UMAP cluster shown in **(B)**.

To identify cell subsets within our high-dimensional data visualized with UMAP, we ran PhenoGraph on the CD45^+^ population (24). PhenoGraph clustering identified 36 populations of lymphocytes (Figure 3A). We labeled the previously generated two-dimensional UMAP projection of CD45^+^ cells by these results and observed that most PhenoGraph populations were found within CD4^+^ and CD8^+^ UMAP clusters. Two populations (#7 and #33) were identified in the cluster between cells marked by high expression of CD4 and CD56 and another two (#10 and #28) were spanning two UMAP clusters (Supplementary Figure 2A). Four out of the 36 populations had a majority of cells (>91%) falling within the CD3^−^CD56^+^ UMAP cluster (Figure 3B left plot and Figure 3D, indicated by the arrows). Displaying all 36 PhenoGraph populations' frequencies as a proportion of the total CD45^+^ population within each individual sample showed a high level of diversity in the lymphocyte repertoire between samples and across tissues analyzed (Figure 3C). The liver was the most heterogeneous, with major differences in the CD4^+^ cluster, but also in the CD3^−^CD56^+^ cluster (Figure 3C). Analysis of PhenoGraph populations within the CD3^−^CD56^+^ UMAP cluster revealed that 3 of them were present only in liver and blood, while population #27 was the only one that was also present in tonsil (Figure 3D).

This analysis shows that PhenoGraph, combined with a dimensionality reduction technique such as UMAP, represents a powerful approach to visualize the general diversity of immune cells within an individual, and to assess tissue distribution of immune cell subsets.

### Detection of Human Liver-Enriched NK Cell Populations in High-Dimensional Space

**Figure 4:**
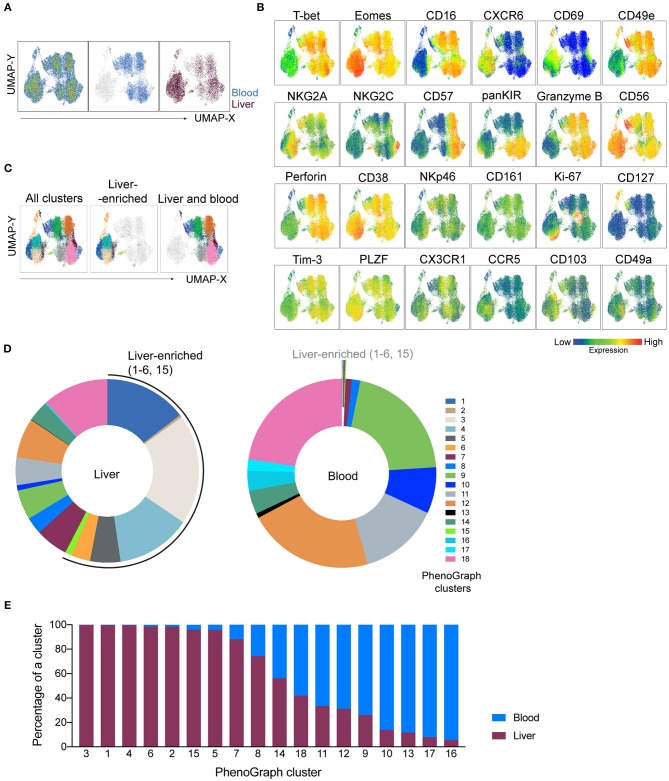
NK-cell focused panel coupled with dimensionality reduction and clustering techniques detects liver-enriched clusters. UMAP and PhenoGraph were run on CD3^−^CD56^+^ UMAP cluster as gated in Figure 3B, containing cells from liver and blood. CD45, DCM/Lin, CD3, and CD4 were excluded from the list of running parameters for UMAP and PhenoGraph. **(A)** UMAP projection of concatenated CD3^−^CD56^+^ cells from non-matched liver (*n* = 6) and blood (*n* = 6) samples, either as a pseudocolor plot combining all samples (left plot) or colored according to the tissue of origin (plots on the right). **(B)** Resulting UMAP embeddings, colored according to the expression of markers from the NK-cell focused panel. **(C)** UMAP embedding from **(A)**, overlaid with 18 identified PhenoGraph clusters. Color-coding is the same as indicated by the legend in **(D)**. **(D)** Donut plots showing frequency of 18 identified PhenoGraph clusters within liver and blood CD3^−^CD56^+^ cells. Liver-enriched clusters are indicated in both sample sources. **(E)** Relative abundance of liver and blood CD3^−^CD56^+^ cells within every detected PhenoGraph cluster.

The scope of this study was to describe the heterogeneity between tissue-resident and circulating NK cells. We showed that NK cells from tonsils contributed to the total number of cells in the CD3^−^CD56^+^ UMAP cluster (Supplementary Figure 2B) and the inclusion of tonsil tissue aided in panel design and validation. However, the NK cell frequency is low in tonsil compared to liver and blood (Supplementary Figure 2C) and our panel and study aim was not to distinguish and analyse tonsil NK cells in relation to other ILC1 populations found in this tissue as this has been reported elsewhere (25). Thus, for the subsequent downstream analysis of NK cells, we focused on liver and peripheral blood and performed further UMAP analysis of these cells (gated as in Figure 3B, left plot). UMAP again separated liver-specific NK cells from the other big cluster of cells shared between blood and liver (Figure 4A). Such clustering appeared to be driven by proteins with specific expression patterns associated with tissue residency (expression of CXCR6 and CD69 but absence of CD49e, T-bet, and CD16) since their expression levels displayed the highest difference between the two big clusters (low-to-high expression) (Figure 4B). Most of the other proteins (i.e., NKG2A, CD38, CD161, Tim-3, PLZF) were expressed at various intermediate-to-high levels in the clusters. (Figure 4B). As before, we next applied PhenoGraph on total liver and peripheral blood NK cells. Eighteen populations were identified, each one with a different contribution to the total population of NK cells in liver and blood (Figures 4C,D). Populations #1, #2, #3, #4, #5, #6, and #15 were most highly enriched in liver over blood (>95% found in liver; Figure 4D). Populations #14 and #18 were present at almost equal frequencies between liver and blood (“shared” clusters). Populations #9, #10, #11, #12, and #13 were found at higher frequencies in blood compared to liver and populations #16 and #17 were almost exclusively detected in blood (Figure 4E). CD127 was highly expressed in a separate cluster connecting CD49e^−^ (liver-enriched) and CD49e^+^ (liver and blood) UMAP clusters.

PhenoGraph revealed the underlying heterogeneity between blood and liver CD3^−^CD56^+^ cells and demonstrated a higher diversity of NK cell subsets in liver when compared to blood, with seven main phenotypes enriched in the liver.

### Assessment of Phenotypic Diversity Within Liver-Enriched NK Cells

**Figure 5:**
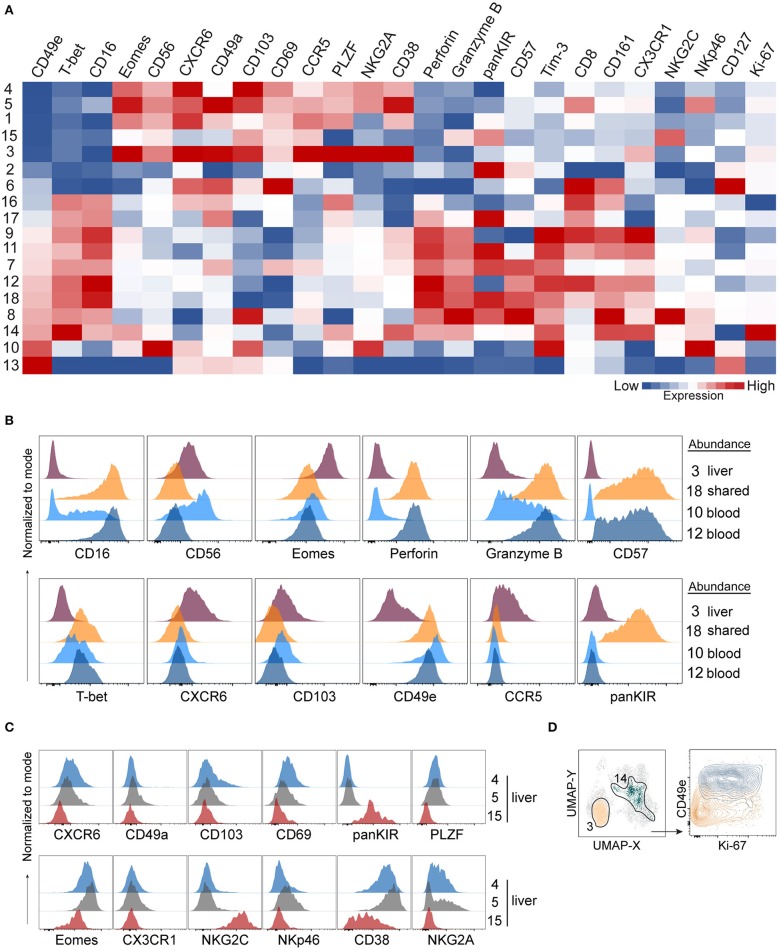
A 29-color NK-cell focused panel captures phenotypic diversity within liver-enriched NK cells. **(A)** Heatmap displays Z-score transformed median expression values for each of the parameters within 18 identified PhenoGraph clusters as described in Figure 4. Color scale was determined for each column separately, based on the lowest and highest Z-score value of that parameter. **(B)** Histograms displaying expression levels of selected proteins in PhenoGraph clusters. Legend indicates whether a cluster is predominantly enriched in liver (#3), present in similar frequencies in liver and blood (shared, #18) or enriched in blood (#10, #12). **(C)** Histograms displaying expression levels of selected proteins from **(A)** in selected liver-enriched clusters (#4, #5, #15). **(D)** Identification of the two most proliferating PhenoGraph clusters (#3 and #14) according to their Ki-67 expression level based on analysis in **(A)**, displayed against CD49e.

Having established that numerous NK cell phenotypes exist in liver and blood and that they differ we next systematically analyzed the phenotype of NK cells identified in the 18 PhenoGraph-derived populations. To this end, we summarized expression levels of all proteins in our 29-color panel for each population (Figure 5A). One of the most differentially expressed proteins between these two subsets was CD49e. In fact, CD49e was the only protein with an expression pattern that reliably recapitulated clustering according to the tissue of origin. This was underlined as sorting of the PhenoGraph clusters according to the increasing levels of CD49e organized the subpopulations similarly to what was obtained by analyzing tissue-enrichment (Figures 4E, 5A). Finally, and in contrast to the other more variable tissue residency markers, CD49e displayed a more uniform expression. Thus, we could recapitulate previously described phenotypes: CD49e^+^ NK cells, predominantly found in blood or in blood and liver, expressed high levels of T-bet, CD16, perforin, and granzyme B, for example (#9, #11, #18). Our panel allowed us for the first time to observe the simultaneous expression of these proteins on the same cell, together with additional markers that also appeared to be differentially expressed between blood and liver. For instance, CD49e^−^ NK cells displayed generally lower levels of Tim-3, CX3CR1, and NKp46. However, even within the CD49e^−^ cells, we observed clusters with a high expression of certain markers typically associated with the CD49e^+^ cells, and vice versa. For example, population #8 was CD103^+^, while population #15 expressed granzyme B. Similarly, CD49a expression was found in two CD49e^+^ populations. One of them was #13, which expressed CD127, several tissue residency markers (CXCR6, CD49a, CD103, CD69) and low-to-none of the conventional NK markers. Together with #6, they appeared to contain the majority of blood and liver ILCs, respectively.

Next, we compared NK cell populations with different tissue origins: one liver-enriched population (#3), one which was present in similar frequencies in blood and liver (“shared,” #18), and two blood-enriched populations, #10 and #12, which appeared phenotypically as CD56^bright^ and CD56^dim^ NK cells, respectively (Figure 5B). The liver-enriched population was different from the other three populations phenotypically, and although #18 and #12 were phenotypically very similar and resembling CD56^dim^ NK cells with respect to CD57, granzyme B, and perforin expression, population #18 was KIR-positive whereas #12 was KIR-negative (Figure 5B).

Finally, we observed a high level of heterogeneity within liver-enriched subsets. Interestingly, variable levels of expression were characteristic of proteins most commonly associated with tissue residency. For example, although populations #4, #5, and #15 were all more common in the liver, #4 and #5 were CXCR6^+^CD103^+^CD69^+^, while #15 was CXCR6^−^CD103^−^CD69^low^. Additionally, only #5 had a low level of CD49a expression, but population #15 was distinguished from the other two by being NKG2C^+^CD38^low^KIR^+^PLZF^−^ (Figure 5C). The only two populations showing clear signs of proliferation, indicated by a high Ki-67 expression, were #3 (liver-enriched, CD49e^−^) and #14 (blood-enriched, CD49e^+^) (Figure 5D).

Together, these data showed that although commonly recognized tissue residency markers in liver NK cells were highly expressed in liver-enriched NK cell subsets compared to blood, they were not uniformly expressed at high levels when a detailed analysis was performed on populations of these cells. By contrast, our analysis found CD49e to robustly separate liver-enriched from blood- and liver-shared NK cell populations.

## Discussion

Flow cytometry is a widely-adopted technology used to investigate the dynamics of immune responses. In the present study, we implemented a 29-color state-of-the-art flow cytometry panel to investigate the diversity of human liver tissue-resident NK cells. The panel encompasses proteins involved in tissue residency, transcription factors, maturation, and effector functions (activating and inhibitory receptors, cytotoxicity potential, activation). We employed a non-linear dimensionality reduction technique to visualize the high-dimensional dataset generated, and used it in conjunction with a clustering approach to detect cellular phenotypes associated with tissue residency. We demonstrate that this approach is robust and can be used to explore NK cell diversity in tissues such as liver and tonsil, but it can also be applied to other organs (e.g., uterus, lung, skin, spleen, salivary gland), with only minor alterations. The analysis framework described here can also be readily adapted to study tissue-resident NK cells in settings of disease where clinical parameters can be included as additional parameters in the analysis.

The technological advances that have led to a significantly increased resolution in the study of single cells brought with them the curse of dimensionality (26). This has been the case with RNA-sequencing methods over the past decade, resulting in the development of many tools for the analysis of high-dimensional data (27). An explosion of bulk and single-cell RNA-sequencing methods has multiple implications for flow cytometry. Firstly, thousands of protein-coding genes and their expression levels can be quantified, and researchers now have more gene candidates than ever to investigate further, including the downstream biological functions of putative proteins. Flow cytometry is the first port of call for such experiments, due to well-established sensitivity and robustness. Secondly, next generation of flow cytometry analyzers has brought the curse of dimensionality into the flow cytometry field. On the other hand, the same tools (or their adaptations) which were developed for RNA-sequencing analysis can also be used in high-dimensional cytometry analysis, such as UMAP used in this study. Thirdly, recently developed methodologies such as CITE-seq and REAP-seq (28, 29) enable concurrent investigation of transcript and protein levels, which can mitigate the shortcomings of RNA-sequencing methods alone, such as weak correlation between the levels of a detected transcript and its translated protein (30–32). Information obtained from these novel experimental pipelines can in powerful ways describe the immune landscape and emphasizes the shift towards the need for high-dimensional flow cytometry.

Along these lines, CyTOF has become a useful tool for immunologists in recent years, since it had emerged as technology enabling investigation of more parameters than possible with conventional flow cytometers at the time (33). While it is a powerful method, CyTOF implementation in the experimental workflow may not always be feasible, depending on the experimental question. Metal isotopes used in CyTOF essentially mitigate compensation-caused issues during data analysis, but cells are destroyed during ionization and cannot be sorted for downstream experiments. Therefore, a flow cytometry panel informed by CyTOF findings still might have to be optimized, should one decide to investigate live cells in downstream applications. Most importantly, flow cytometry is the highest throughput approach in single-cell analysis, as tens of thousands of cells can be run per second, at a low cost of operating (34).

The human NK cell repertoire is highly diverse within and between individuals (7). The conventional classification of NK cells into CD56^bright^ and CD56^dim^ subsets captures only the major differences in the subset-associated phenotypes. However, this is insufficient to explain the different functions that phenotypically similar subsets can exhibit in different tissues. CD56 has an unclear function itself and its “brightness” is not a good discriminator when it comes to implications of surface expression on NK cell functions. CD56^bright^ NK cells are considered to be non-cytotoxic and with immunoregulatory functions, but they can also exhibit enhanced cytotoxicity and degranulation against viral and tumor antigens (35). Along these lines, even absence of CD56 on NK cells marks a specific subset of CD56^neg^ NK cells resembling CD56^dim^ with moderate responsiveness and differential expression of several granule proteins (36, 37). This demonstrates the necessity to assess NK cell phenotypes (and consequently their biological functions) as a set of markers, rather than relying on individual bimodal-expression-based classifications. The panel we designed represents a collection of markers most commonly described to be differentially expressed on liver NK cells. Our findings here substantiate the major findings of previous studies phenotypically describing liver NK cells (38), but also combine them and additionally identify novel differences within liver NK cells.

In more detail, performing dimensionality reduction of CD45^+^ cells data in all three tissues ordered events according to their lineage, instead of the tissue of origin. This was sufficient to assess the general landscape of T cells, non-NK ILCs, and NK cells. However, narrowing the scope of the analysis to the NK cell lineage in liver and blood robustly separated subsets based on the relative enrichment in the tissue and suggested that distinct cellular phenotypes drove this separation. Out of all markers in our panel, CD49e expression most reliably ordered NK cell populations according to their tissue origin, as liver-enriched populations were all CD49-negative, corroborating previous CyTOF findings (19). This suggested that CD49e should be included in future studies of liver-enriched NK cells, and validated the importance of this marker in studies of tissue-resident subsets through another experimental approach. Future work should also address the exact role of CD49e and what the lack of expression means for the function of intrahepatic NK cells. All other liver-enriched populations expressed higher levels of CXCR6, CD49a, CD103, and CD69 as well as CCR5 compared to NK cells enriched in blood or shared between blood and liver.

We took a conservative approach when interpreting our unbiased clustering results in the context of studies that used manual gating to quantify and describe NK cells. Nonetheless, we still detected phenotypic similarities to populations described in those studies. For example, cluster #10 (Figures 4D,E, 5A) appeared to resemble previously described cytokine-induced CXCR6^+^ blood NK cells since this cluster was CD56^bright^CD69^+^ and also expressed higher levels of NKG2C than non-CXCR6^+^ blood-enriched clusters (e.g., cluster #9) (39). An elegant study demonstrated that liver microenvironment TGF-β is required to induce and maintain a liver-resident phenotype (40). However, liver-conditioned media used in that study could not induce CXCR6 on blood NK cells, in contrast to earlier findings with cytokines (39). The panel we propose, addressing tissue resident surface markers as well as transcriptional program associated with acquisition/loss of tissue residency, will be a valuable tool in future studies of how tissue residency is maintained as it aids identification of exact subpopulations in response to dynamic changes in the microenvironment, given the heterogeneity of tissue resident subsets. Our results also corroborate recent findings that liver CXCR6^+^ NK cells contain a high percentage of educated NK cells, considered to be NKG2A^+^ when compared to blood and liver CXCR6^−^ counterpart (41). In our dataset, liver-enriched CXCR6^+^ clusters #3, #4, and #5 are also highly NKG2A^+^, while CXCR6^−^ liver-enriched cluster #2 has a high KIR expression and low levels of NKG2A (Figure 5A). We also identify cluster #15 in the liver with the highest expression of NKG2C and lowest expression of CXCR6, similar to previous studies (41). However, cluster #1 that we identified in liver was CXCR6^+^ but NKG2A^−^ as well as KIR^low^. Our panel could therefore be adapted to investigate the relationships between these clusters in context of education in future studies.

In general, a variable pattern of expression of a majority of tissue residency markers examined calls for caution when interpreting the results, but also suggests the existence of differential gene regulation pathways in distinct liver-enriched clusters. Multiple levels of gene regulation could be analyzed. It would be valuable to obtain transcriptome data for these clusters, for example by performing CITE-seq on single cells with antibodies used in this study, conjugated to oligonucleotides. This information could help determine the relationship between populations we identified here, reveal potential differentiation trajectories, and answer questions such as why some CD49e-negative subsets had low levels of CXCR6. However, since these populations have relatively similar phenotypes, it might be that posttranscriptional gene regulation is more important in regulating the functional potential of various subsets of liver NK cells, in a manner recently suggested to explain increased granzyme B levels in human educated peripheral blood NK cells (42). A limitation of our study is that we used non-matched samples as well as liver samples from non-affected areas of patients undergoing liver resection surgery for primary or secondary tumors. This type of samples has been routinely used in earlier work describing liver-resident NK cell subsets (14, 16, 17). However, it may be that the heterogeneity of NK cell subsets we observed in our study between similarly obtained individual liver samples, for example, originates from a different stage of malignancy and consequently an altered microenvironment between individual donors. Since we demonstrated that our workflow can detect this variability, future studies applying this approach on larger cohorts of patients with well-defined underlying pathologies will be useful in addressing the cause of such variability.

In summary, and in addition to the 29-color panel developed here, we carried out our analysis via a user-friendly interface, although thorough understanding of the nature of high-dimensional data, data transformation methods, and clustering approaches are still required. We used algorithms that minimized bias and maximized unsupervised analysis of the data with caution. Biological knowledge is still essential to avoid data misinterpretation that might originate from algorithms attributing fluorochrome aggregates to rare subsets, for example. Thus, manual analysis is far from obsolete and will remain essential for the foreseeable future (21). In the present study, we examined only six samples per sample source. Regardless, our workflow was robust enough to demonstrate intra- and inter-sample diversity of cellular phenotypes even among these samples. In the future, such a workflow can be applied to large cohorts to give enough statistical power to confidently identify phenotype metaclusters associated with disease states or correlating with other molecular biomarkers. Patient samples usually come with the caveat of limited sample material, and time and simplicity of experimental manipulation are often of essence. Therefore, high-dimensional flow cytometry holds great promise to be a major tool in investigation of complex immune responses, due to the excellent sensitivity and high-throughput nature of the approach. We anticipate similar workflows to the one we describe here to become a routine in investigating NK cells residing in other lymphoid and non-lymphoid organs and the immune responses they are involved in, both during normal homeostasis and in disease.

## Data Availability Statement

The datasets generated for this study are available on request to the corresponding author.

## Ethics Statement

The studies involving human participants were reviewed and approved by the Regional Ethics Committee of Stockholm, Stockholm, Sweden. The patients/participants provided their written informed consent to participate in this study.

## Author Contributions

IF designed the study, performed experiments, acquired and analyzed data, and drafted the manuscript. IS, MC, and LH contributed to the data analysis and the discussion. BS contributed to the data analysis, discussion, and edited the manuscript. DF provided clinical samples. NB and MI designed the study, performed data analysis, edited the manuscript, and supervised the work.

### Conflict of Interest

The authors declare that the research was conducted in the absence of any commercial or financial relationships that could be construed as a potential conflict of interest.
