# Supplementary material for: 29-Color Flow Cytometry: Unraveling Human Liver NK Cell Repertoire Diversity
Source: Front Immunol. 2019 Nov 19;10:2692. doi: 10.3389/fimmu.2019.02692 (PMC6878906; doi:10.3389/fimmu.2019.02692)
Supplement: Supplementary file 1 [file Data_Sheet_1.PDF]

Supplementary Figure 1

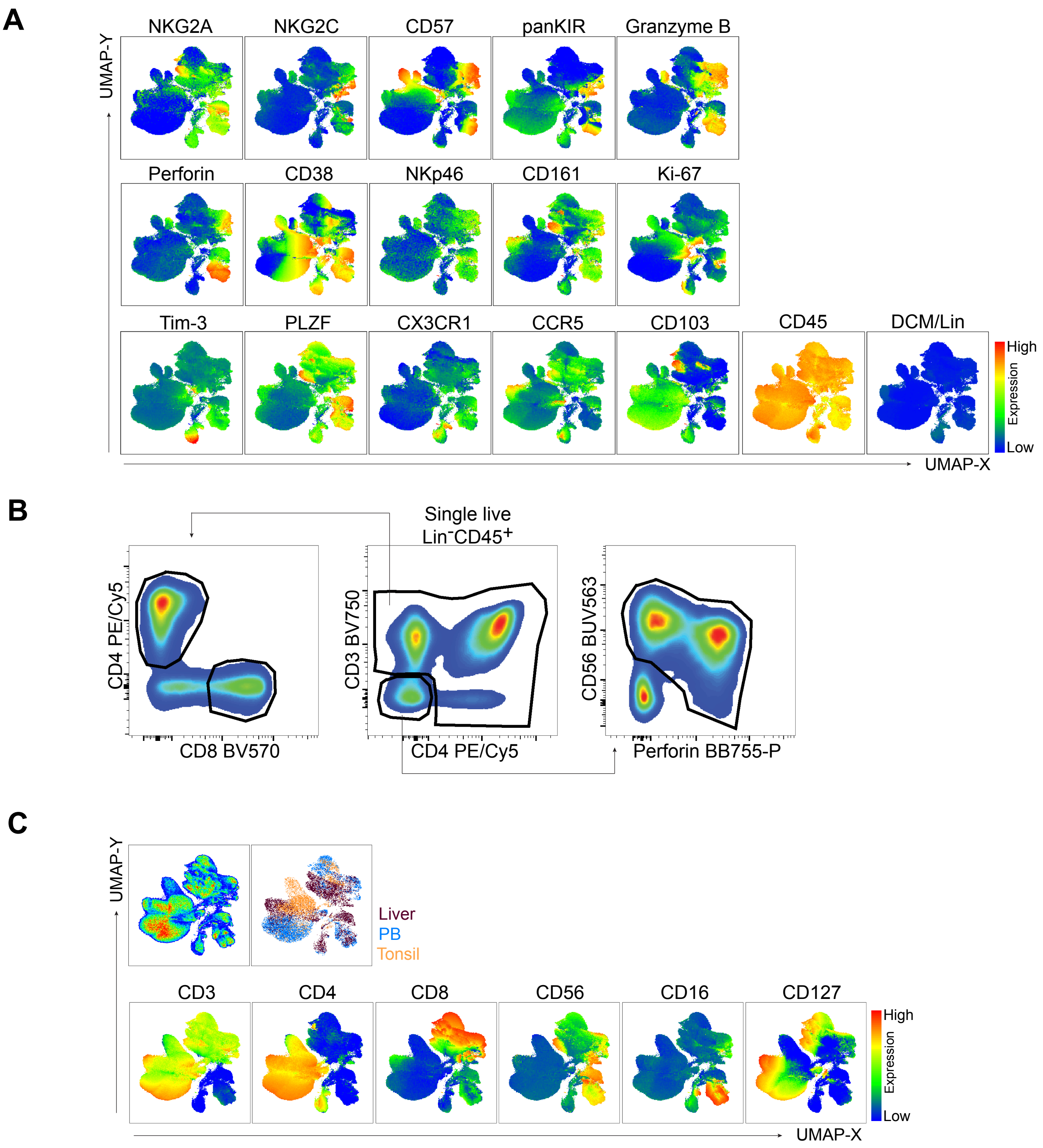

Supplementary Figure 1. T cell-, ILC- and NK cell clusters are separated by non-linear dimensionality reduction. Live, Lin$^-$CD45$^+$ cells were gated and processed as described in **Figure 2**. **(A)** UMAP projection of remaining markers included in the 29-color panel. **(B)** Summary of the representative gating strategy to manually gate NK cells, CD4$^+$ and CD8$^+$ T cells within the concatenated live, Lin$^-$CD45$^+$ population. NK cells (CD3$^-$CD4$^-$CD56$^{bright/dim-}$ Perforin$^{bright/dim}$), CD4$^+$ T cells (CD3$^+$CD4$^+$), CD8$^+$ T cells (CD3$^+$CD8$^+$). **(C)** UMAP projections of the CD45$^+$ cells with CD45, DCM/Lin, and CD56 excluded from the list of running param--eters, showing expression intensities of the indicated markers displayed in **Figure 2B**.

Supplementary Figure 2

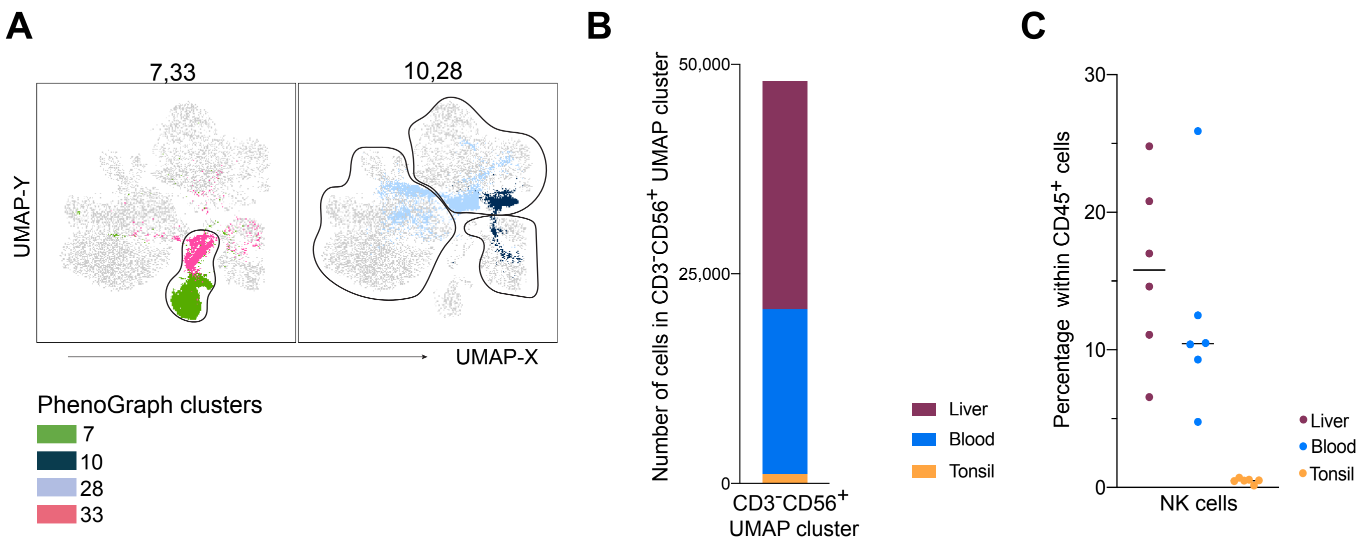

Supplementary Figure 2. PhenoGraph analysis of the Lin$^-$CD45$^+$ population identifies diversity in tissue-enriched clusters. (**A**) Two PhenoGraph clusters displayed in a cluster between CD3$^+$CD4$^+$ and CD3$^-$CD56$^+$ UMAP cluster (#7, #33) and another two PhenoGraph clusters spanning two UMAP clusters each (#10, #28). **(B)** Total number of cells in the CD3$^-$CD56$^+$ UMAP cluster, stratified by the tissue they are found in. **(C)** Percentage of NK cells among single live Lin$^-$CD45$^+$ cells in each tissue analyzed.

.
